# Visualization and Identification of Bioorthogonally Labeled Exosome Proteins Following Systemic Administration in Mice

**DOI:** 10.3389/fcell.2021.657456

**Published:** 2021-04-07

**Authors:** Eric Zhang, Yanwen Liu, Chaoshan Han, Chengming Fan, Lu Wang, Wangping Chen, Yipeng Du, Dunzheng Han, Baron Arnone, Shiyue Xu, Yuhua Wei, James Mobley, Gangjian Qin

**Affiliations:** ^1^Department of Biomedical Engineering, University of Alabama at Birmingham, Birmingham, AL, United States; ^2^Department of Anesthesiology and Perioperative Medicine, University of Alabama at Birmingham, School of Medicine, Birmingham, AL, United States

**Keywords:** exosomes, extracellular vesicles, mesenchymal stem cells, bioorthogonal labeling, click reaction, cardiac ischemia

## Abstract

Exosomes transport biologically active cargo (e.g., proteins and microRNA) between cells, including many of the paracrine factors that mediate the beneficial effects associated with stem-cell therapy. Stem cell derived exosomes, in particular mesenchymal stem cells (MSCs), have been shown previously to largely replicate the therapeutic activity associated with the cells themselves, which suggests that exosomes may be a useful cell-free alternative for the treatment of cardiovascular disorders. However, the mechanisms that govern how exosomes home to damaged cells and tissues or the uptake and distribution of exosomal cargo are poorly characterized, because techniques for distinguishing between exosomal proteins and proteins in the targeted tissues are lacking. Here, we report the development of an *in vivo* model that enabled the visualization, tracking, and quantification of proteins from systemically administered MSC exosomes. The model uses bioorthogonal chemistry and cell-selective metabolic labeling to incorporate the non-canonical amino acid azidonorleucine (ANL) into the MSC proteome. ANL incorporation is facilitated via expression of a mutant (L274G) methionyl-tRNA-synthetase (MetRS^∗^) and subsequent incubation with ANL-supplemented media; after which ANL can be covalently linked to alkyne-conjugated reagents (e.g., dyes and resins) via click chemistry. Our results demonstrate that when the exosomes produced by ANL-treated, MetRS^∗^-expressing MSCs were systemically administered to mice, the ANL-labeled exosomal proteins could be accurately and reliably identified, isolated, and quantified from a variety of mouse organs, and that myocardial infarction (MI) both increased the abundance of exosomal proteins and redistributed a number of them from the membrane fraction of intact hearts to the cytosol of cells in infarcted hearts. Additionally, we found that Desmoglein-1c is enriched in MSC exosomes and taken up by ischemic myocardium. Collectively, our results indicate that this newly developed bioorthogonal system can provide crucial insights into exosome homing, as well as the uptake and biodistribution of exosomal proteins.

## Introduction

The therapeutic benefits of stem cells are mediated primarily via the secretion of paracrine factors (Bagno et al., 2018), and recent evidence suggests that the exosomes produced by mesenchymal stem cells (MSCs) largely replicate the improvements associated with administration of the cells themselves in large-mammal models of myocardial injury (Charles et al., 2020). Thus, while exosomes could be a useful cell-free alternative to MSC therapy, little is known of how exosomes are directed toward injured tissue, the distribution of the exosomal cargo in target organs and cells, or the physiological mechanisms that are induced by exosome uptake. This information is crucial for identifying the appropriate dose, timing, and route of administration for exosome therapy but remains difficult to decipher, as techniques for tracking exosomes and the exosome cargo are limited. Radiolabeled exosomes have been used to determine the half-life of exosomes in the circulation and to identify some of the major sites of exosome uptake (Morishita et al., 2015), but they cannot track the presence of exosome proteins in target organs and are dependent on half-life of the radiolabel. Thus, methods for accurately and reliably identifying both the source and targets of the exosomal cargo in living organisms will dramatically advance exosome research (Mathieu et al., 2019).

Cell-selective metabolic labeling generates proteins containing non-canonical amino acids with functional groups that can be linked to affinity reagents or fluorescent dyes for subsequent identification, isolation, and imaging. It was previously shown that the mutant *E. coli* methionyl-tRNA synthetase (MetRS^L274G^ or MetRS^∗^) selectively appends the azide-bearing non-canonical amino acid azidonorleucine (ANL) to tRNA. Thus, when cells that express MetRS^∗^ are incubated with ANL, they produce proteins that contain ANL in lieu of endogenous methionine (Ngo et al., 2009) and can be covalently linked to alkyne-tagged reagents (e.g., dyes and resins) via click chemical reactions, such as copper-catalyzed azide-alkyne cycloaddition. These non-canonical amino acids are not produced *in vivo* and as such are excluded during normal protein synthesis.

For this study, we used non-canonical amino-acid labeling to develop an *in vivo* model that enables us to visualize, track, and measure the biodistribution of proteins from systemically administered MSC exosomes. MSCs were transfected with a lentiviral vector coding for MetRS^∗^ and incubated with ANL-supplemented media; then, exosomes were isolated from the MSC-conditioned media and intravenously administered to mice. Our results demonstrate that the ANL-labeled exosomal proteins can be accurately and reliably identified, isolated, and quantified from a variety of mouse organs, and that surgically induced myocardial infarction (MI) both increased the abundance of labeled exosomal proteins and redistributed the localization from the membrane to the cytosol within organs following systemic administration.

## Materials and Methods

### MSC Culture and Characterization

Mouse (C57BL/6) bone marrow MSCs were purchased from Cyagen Biosciences (Sunnyvale, CA, United States, Catalog Number: MUBMX-01001), cultured in Dulbecco modified Eagle medium (DMEM) supplemented with 10% fetal bovine serum (FBS), and passaged 5–8 times before use. MSC identity was a confirmed via flow-cytometry analyses of MSC surface marker expression and assessments of osteogenic and adipose differentiation potential. Differentiation assays were performed by following the manufacturer’s protocols (Cyagen Biosciences, United States). Briefly, the cells were cultured on 0.1% gelatin-coated six-well plates (2 × 10^4^ cells/cm^2^) in complete medium until confluent and then treated with osteogenic differentiation medium for 3 weeks or with adipocyte induction medium and maintenance medium for 2–3 weeks; osteogenic and adipose differentiation were analyzed by Alizarin staining and by Oil Red O staining (for lipid droplets), respectively.

### Flow Cytometry

Flow cytometry was performed as previously described (Cheng et al., 2019). Briefly, 1 × 10^6^ cells were incubated in 200 μL cold phosphate-buffered saline (PBS) with Fc block (1:100 dilution), washed three times, stained with phycoerythrin (PE)-conjugated antibodies for mouse CD34 (1:50) or CD44 (1:50), or with fluorescein isothiocyanate (FITC)-conjugated antibodies for mouse CD11b (1:50), CD45 (1:50), or Sca-1 (1:50) (ebioscience, Germany). Data were acquired with a BDTM LSR II (BD Biosciences, United States) and analyzed with Cell Quest Software (Becton Dickinson, United Kingdom).

### qRT-PCR

Quantitative reverse transcription PCR (qRT-PCR) was performed as previously described (Tang et al., 2009). Briefly, total MSC RNA was reverse transcribed with random hexamer primer, and 25 ng of the reverse-transcribed product was used for RT-PCR; results with the random primer were consistent with those obtained previously with a primer common to the 3′ untranslated region (UTR) of Dsg1-a,b, and -c. Values were normalized to equivalent assessments of glyceraldehyde phosphate dehydrogenase (GAPDH) mRNA abundance.

### Western Blotting

For protein extraction, 1 × 10^7^ cells or 100 mg of frozen tissue were homogenized in 1 mL RIPA lysis buffer containing protease inhibitors (Sigma, 4693132001) and phosphatase inhibitors (Sigma, 4906837001). Samples were incubated with agitation for 30 min at 4°C and centrifuged at 13,000 rpm for 10 min at 4°C; then, the protein concentration in the supernatant was determined via bicinchoninic acid (BCA) assay (Pierce). For immunoblotting, proteins in the supernatant were denatured by heating at 95°C for 10 min, separated by SDS-PAGE, and then transferred onto a polyvinylidene difluoride (PVDF) membrane (Bio-Rad). The membrane was incubated in 5% nonfat milk blocking buffer (tris-buffered saline [TBS]) for 1 h, incubated with primary antibody in TBS-containing 3% bovine serum albumin (BSA) overnight at 4°C, washed three times with TBS (0.5% Tween 20), incubated with secondary antibody, washed with TBS Tween 20, and then developed with Enhanced Chemiluminescence Detection Reagents (ECL, Thermo Fisher). Protein signals were imaged with a Bio-Rad ChemiDoc System.

### Generation of Lenti-CAG-MetRS^L274G^–mCherry Vector

The Lenti-CAG-MetRS^L274G^–mCherry vector was newly engineered in our lab by using a pLenti.CAG.H2B-Dendra2.W (Addgene #51005) backbone and the MetRS^L274G^–mCherry coding sequence from pMaRSC (Addgene #89189). Briefly, pLenti.CAG.H2B-Dendra2.W was amplified via high-fidelity PCR with 5′ CAG-WPRE-5 primers (containing a sequence complementary to the WPRE region of the vector sequence with an Asc1-Age1-Mlu1-EcoRV restriction-site overhang) and 3′ CAG-LINK-3 primers (containing a sequence complementary to the Sal1 and upstream region of the vector sequence with an EcoRV-Mlu1-Age1-Asc1 restriction-site overhang). The 7.9-kb PCR product, which included the majority of the pLenti.CAG.H2B-Dendra2.W sequence but lacked the 1.1-kb H2B-Dendra2.W-coding region, was transformed into Top 10 competent cells (Thermo Scientific), where it was circularized via self-assembly cloning (Matsumoto and Itoh, 2011) into a pLenti.CAG.linker (containing multi-cloning sites Sal1-Asc1-Age1-Mlu1-EcoRV) and verified by Mlu1 enzyme digestion. To clone the MetRS^L274G^–mCherry coding sequence, PCR was performed with 5′ Sal1-L274G-mCherry 5 primers, 3′ Age1-L274G-mCherry 3 primers, and the pMaRSC template (Addgene #89189) for 20 cycles; each cycle consisted of 30 s at 95°C, 30 s at 60°C, and 4 min at 72°C. The 4-kb PCR product, which contained the MetRS^L274G^-mCherry coding sequence, was subsequently cloned into TA cloning vector PCR^TM^ 2.1 (Thermo Scientific), and positive clones were verified by digestion with Sal1 and Age1; then, the 4-kb MetRS^L274G^-mCherry fragment was recovered with agarose gel and ligated into the Sal1/Age1-digested pLenti.CAG.linker to produce pLenti.CAG.MetRS^L274G^-mCherry, which was confirmed by DNA sequencing. The Lenti.CAG.MetRS^L274G^-mCherry vector was produced by co-transfecting pLenti.CAG.MetRS^L274G^-mCherry with pMD2.G and psPAX2 into 293FT cells, and then purified and concentrated as previously described (Cheng et al., 2010). All primer sequences are reported in Supplementary Table 1.

### Lentivirus Transduction and ANL Labeling of Nascent Cellular Proteins

For lentivirus transduction, MSCs were seeded at ∼80% confluence, and the lentivirus (MOI = 4) was applied with polybrene (8 μg/mL); transduction efficiency was evaluated 72 h later by monitoring mCherry fluorescence with a fluorescence microscope or via fluorescence-activated cell-sorting (FACS); control assessments were conducted with MSCs that had been transduced with Lenti.CAG.H2B-Dendra2.W. For ANL labeling, cells were washed twice in PBS and then suspended in methionine-, glutamine-, and cystine-depleted DMEM (Gibco, Cat. #21013024) containing 1 mM ANL (IRIS Biotech, Cat. #159610-92-1); the DMEM had been made complete with glutamine and cystine and supplemented with exosome-free FBS (Gibco, Cat. #A270801). Cells were cultured for 72 h before use in subsequent experiments.

### Bio-Orthogonal Non-canonical Amino Acid Tagging (BONCAT)

MetRS^∗^-transduced MSCs were incubated in 1 mM ANL for 24 h and lysed in RIPA buffer supplemented with protease inhibitors (Sigma, United States); then, total protein was collected, and the Click-iT reaction was performed with a Click-iT Protein Reaction Buffer Kit (Invitrogen, Waltham, MA, United States) as directed by the manufacturer’s instructions. Briefly, up to 200 μg of ANL-labeled protein was reacted with 100 μL of Click-iT reaction buffer containing alkyne-Cy7 and copper-sulfate catalyst in a rotator for 20 min. Methanol and chloroform were added to remove residual reaction components and precipitate the proteins; then, the precipitated proteins were solubilized in Laemmli buffer and loaded for gel electrophoresis. Gels were immediately imaged with a ChemiDoc MP Imaging System (Bio-Rad, California, United States) under both stain-free mode and Cy7 mode.

### Fluorescent Non-canonical Amino-Acid Tagging

For fluorescent non-canonical amino-acid tagging (FUNCAT) of MetRS^∗^-transduced MSCs *in vitro*, MSCs were cultured in 1 mM ANL (Jena Bioscience, Jena, Germany) for 24 h, gently rinsed with PBS, fixed in 4% formaldehyde/PBS and permeabilized with 0.5% Triton X-100/PBS; then, the Click-iT reaction was performed with Click-iT reaction cocktails containing Alexa Fluor 488 alkyne (Click-iT Alexa Fluor 488 Protein Synthesis HCS Assay kit, Life Technologies, Waltham, MA, United States) as directed by the manufacturer’s protocol. Cells were stained with Hoechst 33342 prior to imaging. For FUNCAT of MetRS^∗^ exosomal proteins *in vivo*, the general methodology was adapted from previous studies (Ngo et al., 2013). Briefly, mice were euthanized 2 h after exosome administration; then, the hearts were explanted, perfused, fixed by incubation in 4% paraformaldehyde at room temperature for 4 h, cryoprotected in 30% sucrose/PBS solution overnight, frozen in OCT compound, cut into 5-μM-thick sections, and stored at −20°C. Frozen sections were incubated at room temperature for 30 min, washed once with PBS, permeabilized in 0.25% Triton/PBS for 35 min, washed in PBS (3 washes, 5 min each), blocked in commercial blocking buffer (Thermo Scientific, TA-125-PBQ) for 7 min, and washed in PBS again (3 washes, 5 min each); then, the ANL-labeled proteins were conjugated to biotin-alkyne by incubating the sections at 4°C overnight in a 1:10,000 dilutio of biotin-alkyne in 2× Catalyst solution from the Click-iT Enrichment kit (Invitrogen, Cat. #C10416). Sections were thoroughly washed in PBS (5 washes, 5 min each) to remove residual biotin, sequentially incubated with primary biotin antibodies (1:1000 dilution) at 4°C overnight and with secondary antibodies at 4°C overnight, washed PBS (three times), incubated with Sudan Black for 30 min, and then washed again in PBS (three times).

### Isolation and Characterization of MSC Exosomes

^MetRS*^MSCs were plated at ∼50% confluence in complete DMEM for 24 h, after which they were cultured in methionine-depleted DMEM supplemented with 1 mM ANL for 72 h. Conditioned media was collected from the MSCs, spun at 1,000 G to remove cell debris, passed through a 0.22-micron filter, and ultracentrifuged at 120,000 *G* for 2 h; then, the supernatant was removed and replaced with cold PBS, and the exosomes were ultracentrifuged at 120,000 *G* for 2 h. Exosome pellets were resuspended in 10 mL of cold PBS and stored at −80°C until use in subsequent experiments. The size and quantity of freshly isolated exosomes was estimated via nanoparticle-tracking analyses (NanoSight), and morphological assessments were conducted via electron microscopy as previously described (Cheng et al., 2019).

### Mouse MI Model and Intravenous Exosome Delivery

All animal experiments in this report were approved by the Institutional Animal Care and Use Committee (IACUC) of the University of Alabama at Birmingham and performed in compliance with the National Institutes of Health (NIH) publication *Guide for the Care and Use of Laboratory Animals*. Twelve- to 16-week-old C57BL/6J mice (Jackson Laboratory, Bar Harbor, ME, United States) were used for all experiments unless otherwise specified. For surgically induced MI, mice were intubated and connected to a ventilator, and anesthesia was initiated and maintained with inhaled 2% isoflurane USP (Fluriso^TM^, VetOne). The heart was exposed via left thoracotomy, and MI was induced by permanently ligating the left-anterior descending (LAD) coronary artery, as previously described (Qin et al., 2006); sham-operated animals underwent the identical surgical procedure, except LAD artery ligation was omitted. After chest closure, buprenorphine hydrochloride (0.1 mg/kg, Buprenex, Reckitt Benckiser Pharmaceuticals Inc.) and carprofen (5 mg/kg, Rimadyl, Zoetis) were administered via intraperitoneal injection every 12 h for 3 days. Exosomes (10 × 10^9^ in 200 μL PBS) were delivered via tail-vein injection 24 h after MI or Sham surgery, and mice were euthanized via CO_2_ inhalation.

### Isolation of ANL-Labeled Proteins From Whole Organs (Click-Catalyzed Alkyne-Agarose Capture)

Animals were euthanized via CO_2_ inhalation and perfusion with 10 mL cold PBS; then, the heart, brain, lung, kidney, liver, and spleen were isolated, cut into ∼20-mg sections, rinsed twice in PBS, and flash frozen with liquid nitrogen. Membrane and cytosolic total protein fractions were isolated with a Mem-PER Plus Membrane Protein Extraction kit (Thermo Scientific, Cat. #89842), precipitated via methanol-chloroform extraction, and stored at −80°C. For ANL-labeling and MetRS^∗^ protein capture, fractions were resolubilized in Click-iT buffer solution and processed as directed by the Click-iT Protein Enrichment Kit protocol (Invitrogen, Cat. #C10416). Captured peptides were stored at −80°C until identification via liquid chromatography mass spectrometry (LC-MS). To collect protein samples from non-administered exosomes, ∼1 × 10^9^ exosomes were suspended in 100 μL PBS and prepared in parallel with organ samples for LC-MS identification.

### Sample Preparation and Data Acquisition by Liquid Chromatography Mass Spectrometry

Dried peptides were reconstituted in 16 μL of 0.1% formic acid (FA); then, 8 μL of each sample was injected onto a 1,260 Infinity nano high-performance liquid chromatography (nHPLC) stack (Agilent Technologies, Santa Clara, CA, United States), and peptides were separated on a 75-micron (internal diameter) by 15-cm pulled tip C-18 column (Jupiter C-18 300 Å, five micron, Phenomenex) that was attached to a Thermo Orbitrap Velos Pro hybrid mass spectrometer equipped with a nano-electrospray source (Thermo Fisher Scientific, Waltham, MA, United States). All data were collected in CID mode, and the nHPLC was configured with binary mobile phases consisting of solvent A (0.1%FA in ddH2O) and solvent B (0.1% FA in 15% ddH_2_O / 85% ACN) programmed as follows; 10 min at 2% solvent B; 90 min at 5–40% solvent B; 5 min at 70% solvent B; and 10 min at 0% solvent B. After each parent ion scan (300–1,200 m/z; 60 k resolution), fragmentation data (MS2) was collected for the 15 most-intense ions. For data dependent scans, charge state screening and dynamic exclusion were enabled with a repeat count of two, repeat duration of 30 s, and exclusion duration of 90 s.

### MS Data Conversion and Searches

XCalibur RAW files were collected in profile mode, centroided, and converted to MzXML with ReAdWv.3.5.1; then, the mgf files were created by using MzXML2Search (included in TPP v. 3.5) for all scans. Data was searched via SEQUEST, which was set for two maximum missed cleavages, a precursor mass window of 20 ppm, trypsin digestion, and variable modifications C and M at 57.0293 and 15.9949, respectively. Searches were performed with a specific subset of the UniRef100 database.

### Peptide Filtering, Grouping, and Quantification

The lists of peptide identifications (IDs) generated via SEQUEST (Thermo Fisher Scientific, Waltham, MA, United States) were filtered with Scaffold (Protein Sciences, Portland, Oregon, United States). Scaffold generates and retains only high-confidence IDs while producing normalized spectral counts across all samples, and the spectral counts can subsequently be used for relative quantification. Only peptides with charge states ≥2+, minimum lengths of six amino acids, and nonzero quantities for all six mass tags were analyzed. For large datasets, Scaffold incorporates the two most common methods for statistical validation: false discovery rate (FDR), and protein probability; FDR was set at <1% cutoff, individual peptide probabilities were ≥0.8, protein probabilities were ≥0.99, and at least two peptides were assigned per protein. Relative quantification was performed via spectral counting and spectral count abundances were normalized between samples.

### Bioinformatics and Statistics Analysis

For protein abundance data, the Pearson coefficient was calculated to evaluate correlations in protein expression between samples. Hierarchical clustering of samples and protein profiles was based on the complete linkage method, and differences in protein expression between the MI and Sham groups was evaluated with the Mann Whitney *U*-test. Differentially expressed proteins (DEPs) were defined as those with nominal *p*-value < 5% and fold-change >1.5, and were visualized via heatmaps. For proteins that were detected in ≥60% of one group and absent in the other group, we used the method developed by Kojima et al. (2012), with a more stringent cutoff (>60% in only one group); proteins meeting this criteria were defined as “all-or-nothing” hits and included in the list of DEPs.

## Results

### MetRS^∗^ Expression in MSCs Facilitates ANL-Labeling of Nascent MSC Proteins

Murine MSCs were obtained from a commercial vendor, and the characteristic pattern of MSC marker expression (i.e., positive for Sca-1 and CD44; negative for CD11b, CD45, and CD34) was verified via flow-cytometry (Supplementary Figure 1A). Phase-contrast microscopy confirmed that the cells were uniformly spindle-shaped, and robust multipotency was verified by differentiating the cells into adipocytes and osteocytes (Supplementary Figures 1B,C). To facilitate non-canonical amino acid tagging, MSCs were transfected with our newly generated MetRS^∗^ lentiviral vector (Lenti-CAG-MetRS^L274G^-mCherry). The vector also coded for mCherry, which enabled stably transfected cells (^MetRS*^MSCs) to be selected via flow cytometry for mCherry fluorescence, and proteins produced by the ^MetRS*^MSCs were labeled with ANL by incubating the selected cells in 1 μM ANL-supplemented media. Control assessments were conducted with MSCs that had been transfected with an equivalent vector that lacked the MetRS^L274G^-mCherry cassette (^CTL^MSCs).

**FIGURE 1:**
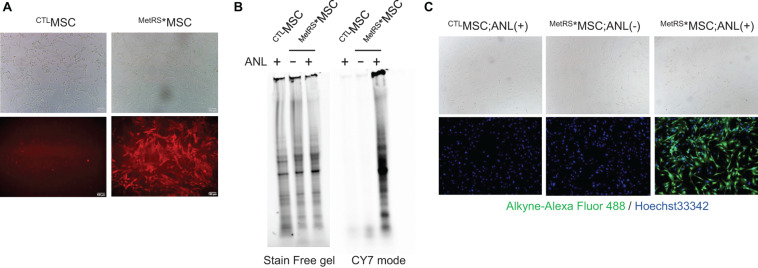
Proteins from ANL-treated ^MetRS*^MSCs can be detected via alkyne-Cy7 click-staining. MSCs were transfected with Lenti-CAG-MetRS^L274G^–mCherry (^MetRS*^MSCs) or a control lentiviral vector (^CTL^MSCs). **(A)** Morphology of the transfected cells was evaluated via phase contrast microscopy (top), and transfection of ^MetRS*^MSCs was confirmed by visualizing mCherry fluorescence (bottom). **(B,C)** ^MetRS*^MSCs and ^CTL^MSCs were incubated with ANL (+) or vehicle (–) for 24 h. **(B)** MSCs were lysed; then, proteins were separated on an SDS-PAGE gel, click-stained with alkyne-Cy7, and imaged in stain-free and Cy7 mode (BONCAT). **(C)** MSCs were permeabilized, click-stained with alkyne-Alexa Fluor 488, and viewed via phase contrast microscopy (top) or under fluorescence (bottom) (FUNCAT); nuclei were counterstained with Hoechst, and ANL-labeled proteins were identified via alkyne-Alexa Fluor 488 fluorescence.

Widespread mCherry expression was observed after just a single transfection (Figure 1A), and there were no apparent morphological differences between ^MetRS*^MSCs and ^CTL^MSCs after 72 h of incubation in the ANL-supplemented media. To confirm that ANL was incorporated into the ^MetRS*^MSC proteome, BONCAT was conducted with lysates from ANL-treated ^MetRS*^MSCs and ^CTL^MSCs: proteins in the lysate were separated on a gel and covalently stained with alkyne-conjugated Cy7. Overall protein levels in the two MSC populations were equivalent; however, the alkyne-Cy7 stain was only observed in proteins from ^MetRS*^MSC lysates, which spanned a wide spectrum of masses (Figure 1B). The results from our BONCAT assessments were also corroborated in intact cells via FUNCAT: the cells were permeabilized, and then click reactions were conducted with a cocktail containing Alexa Fluor 488 alkyne. The fluorescent signal (i.e., ANL-incorporated protein) was highly abundant in the cytosol of ^MetRS*^MSCs but completely absent in ^CTL^MSC cytosol (Figure 1C).

### ANL-Alkyne Click Reactions Detect ANL-Labeled Proteins From ^MetRS*^MSC Exosomes With High Sensitivity and Specificity

**FIGURE 2:**
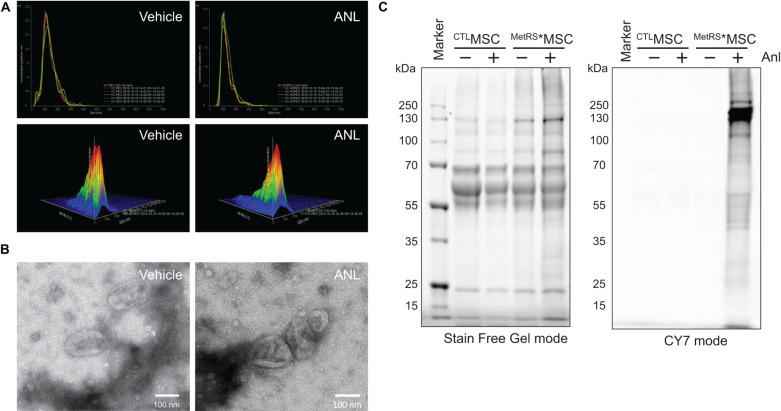
Exosomal proteins from ANL-treated ^MetRS*^MSCs can be detected via alkyne-Cy7 click-staining. **(A,B)** The ^MetRS*^MSCs were incubated with ANL (+) or vehicle (–) for 24 h; then, exosomes were isolated from culture media and evaluated via **(A)** nanoparticle-tracking analysis and **(B)** transmission electron microscopy (TEM). **(C)** Exosomes were isolated from ^CTL^MSCs and ^MetRS*^MSCs that had been incubated with ANL (+) or vehicle (–) for 24 h; then, the exosomes were lysed, and proteins were separated on an SDS-PAGE gel, click-stained with alkyne-Cy7, and imaged in stain-free and Cy7 mode (BONCAT).

Assessments via nanoparticle-tracking analysis (Figure 2A) and transmission electron microscopy (Figure 2B) confirmed that treatment with ANL-supplemented media (1 μM for 72 h) did not substantially alter the production, size, and ultrastructure of exosomes produced by ^MetRS*^MSCs. Furthermore, when exosomes were collected from ^MetRS*^MSCs (i.e., ^MetRS*^MSC-Exos) and ^CTL^MSCs (^CTL^MSC-Exos) that had been grown in media supplemented with or without ANL, BONCAT assessments of their protein cargo indicated that the total amount of protein in all four groups was similar, but alkyne-Cy7 fluorescence was only detected for proteins in exosomes from ANL-treated ^MetRS*^MSC-Exos (Figure 2C). Thus, ANL labeling and subsequent detection was both sensitive and specific for proteins in ^MetRS*^MSC-Exos.

### ANL-Alkyne Click Reactions Detect ANL-Labeled Proteins From Systemically Administered ^MetRS*^MSC-Exos in the Organs of Mice

**FIGURE 3:**
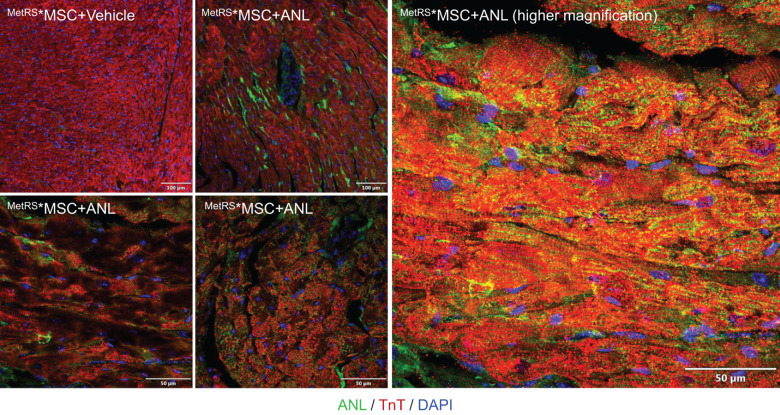
Proteins from systemically administered ^MetRS*^MSC-Exos can be visualized in mouse hearts via click-catalyzed alkyne-Alexa Fluor 488 staining. Exosomes were isolated from the culture media of ANL- or vehicle-treated ^MetRS*^MSCs and injected into the tail vein of mice. Hearts were explanted 2 h later, sectioned, click-stained with reaction cocktails containing Alkyne-conjugated Alexa Fluor 488 (green), and counterstained with anti-Troponin T (red) and Hoechst (blue); regions of overlapping Alexa Fluor 488 and Troponin T fluorescence appear yellow.

To confirm that our method for detecting ANL-labeled ^MetRS*^MSC-Exo proteins could effectively monitor the trafficking of proteins from systemically administered ^MetRS*^MSC-Exos in vivo, exosomes were isolated from ANL- or vehicle-treated ^MetRS*^MSCs and intravenously delivered into mice (20 μg/mouse); 2 h later, cardiac tissues were harvested, sectioned, and analyzed via FUNCAT. The fluorescent signal was strongly observed in both the vasculature and cardiomyocytes of mice that received the ANL-treated ^MetRS*^MSC-Exos but was completely absent in sections from mice that were administered vehicle-treated ^MetRS*^MSC-Exos (Figure 3).

Having confirmed the fidelity of click-catalyzed ANL-staining in our *in vivo* model, we investigated whether ANL-labeled exosome proteins could be isolated from the organs of mice after ^MetRS*^MSC-Exo administration. Proteins were precipitated from whole mouse organs (heart, kidney, spleen, lung, liver, and brain), and then ^MetRS*^MSC-Exo proteins (i.e., proteins containing the ANL-label) were captured from the precipitate with an agarose resin containing alkyne functional groups that, when catalyzed via click reaction, formed covalent links with the ANL label. Because the ^MetRS*^MSC-Exo proteins comprised only a very small fraction of the total amount of protein in each organ, we confirmed the specificity and accuracy of click-catalyzed alkyne-agarose capture by comparing the data for each individual ^MetRS*^MSC-Exo protein to data for the same protein in the corresponding organs from ^MetRS*^MSC-Exo–treated animals when the click catalysis reaction was omitted and from animals that were not treated with ^MetRS*^MSC-Exos.

**FIGURE 4:**
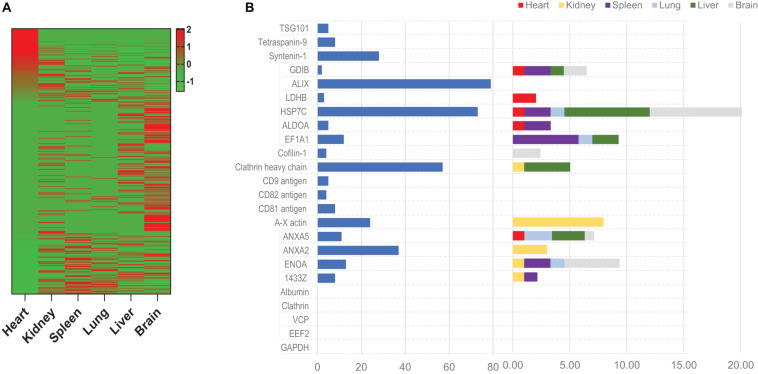
Proteins from systemically administered ^MetRS*^MSC-Exos can be detected, identified, and quantified in mouse organs via click-catalyzed alkyne-agarose capture. ^MetRS*^MSC-Exos were injected into the tail vein of mice. Animals were euthanized 24 h later, and protein extracts were prepared from the heart, kidney, spleen, lung, liver, and brain; then, proteins from the ^MetRS*^MSC-Exos (i.e., those containing the ANL label) were isolated via click-catalyzed alkyne-agarose capture and identified via LC-MS. **(A)** ^MetRS*^MSC exosomal proteins isolated from the indicated organs (average total spectra) were presented as a heat map. **(B)** The abundance of canonical positive and negative exosome marker proteins was quantified in ^MetRS*^MSC-Exos before injection (left) and in the indicated organs of mice after ^MetRS*^MSC-Exo injection (right).

LC-MS analysis identified 205 ^MetRS*^MSC-Exo proteins that were isolated from the hearts of ^MetRS*^MSC-Exo–treated mice exclusively via click-catalyzed alkyne-agarose capture, compared to 29 proteins that were captured only when the click reaction was omitted, and 48 that were isolated both with and without click catalysis (Figure 4A); the abundance of each individual ^MetRS*^MSC-Exo protein varied widely among organs (Figure 4B). Notably, most (but not all) of the exosome-marker proteins that were present in ^MetRS*^MSC-Exos before administration (Supplementary Table 2) were also isolated from at least one of the organs of ^MetRS*^MSC-Exo–treated mice, but they were not equally distributed across all organs (Figure 4C).

### MI Increases Cardiac Uptake and Alters the Subcellular Distribution of ANL-Labeled ^MetRS*^MSC-Exo Proteins

**FIGURE 5:**
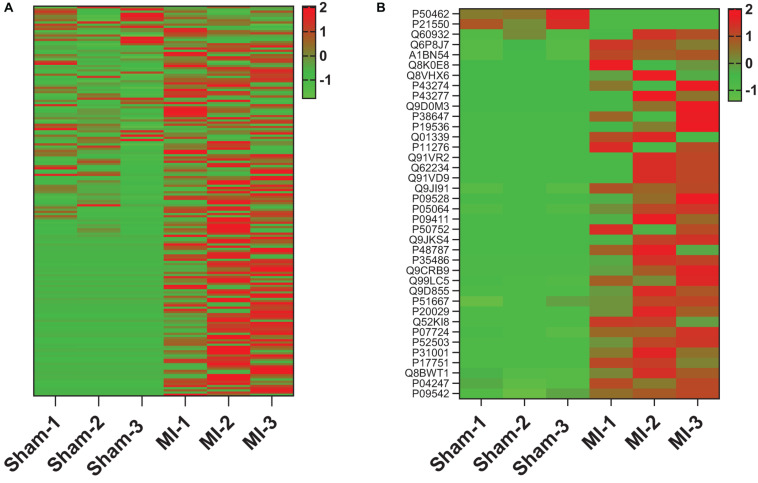
MI increases the abundance of ^MetRS*^MSC exosomal proteins in the heart. ^MetRS*^MSC-Exos were injected into the tail vein of mice 24 h after MI induction or Sham surgery (*n* = 3 per group). Animals were euthanized 24 h after exosome administration, and protein extracts were prepared from the left ventricle; then, the ANL-labeled ^MetRS*^MSC exosomal proteins were isolated via click-catalyzed alkyne-agarose capture and identified via LC-MS. **(A)** Heat map of all identified ^MetRS*^MSC exosomal proteins. **(B)** Heat map of ^MetRS*^MSC exosomal proteins that were differentially expressed in MI and Sham hearts.

**FIGURE 6:**
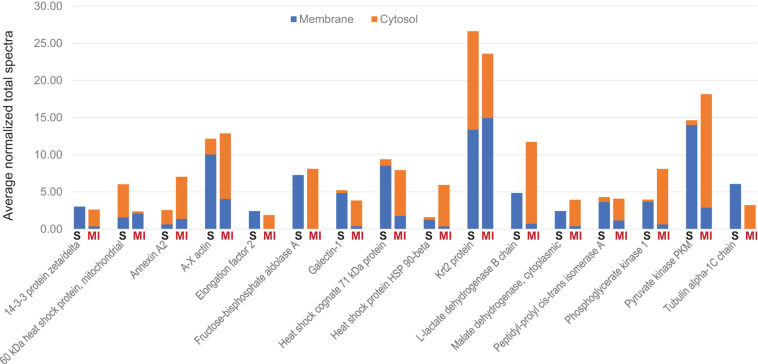
MI alters the subcellular distribution of proteins from systemically administered ^MetRS*^MSC-Exos. ^MetRS*^MSC-Exos were injected into the tail vein of mice 24 h after MI induction or Sham surgery. Animals were euthanized 12 h after exosome administration, and protein extracts from the left ventricle were separated into membrane and cytosolic fractions; then, the ANL-labeled ^MetRS*^MSC exosomal proteins in each fraction were isolated via click-catalyzed alkyne-agarose capture and identified via LC-MS. The abundance of each of the 16 selected canonical exosomal proteins in the membrane and cytosol fractions of hearts from Sham (S) and MI animals was expressed as normalized average spectra from 3 independent samples.

To determine whether tissue injury changes the biodistribution of MSC exosomes, ANL-labeled ^MetRS*^MSC-Exos were administered to mice via tail-vein injection 24 h after surgically induced MI (the MI group) or sham surgery (the Sham group). Cardiac tissue was collected 12 h after ^MetRS*^MSC-Exo administration, and ^MetRS*^MSC-Exo proteins were isolated via click-catalyzed alkyne agarose capture. The number of ^MetRS*^MSC-Exo proteins was dramatically greater in hearts from MI than from Sham animals (Figure 5A), and of those that were present in the hearts of both groups, nearly all were more abundant in MI hearts (Figure 5B). These observations are consistent with the MI-induced uptake of MSC exosomes in cardiac cells. Furthermore, when the membrane and cytosol fractions were evaluated separately, a number of ^MetRS*^MSC-Exo proteins that were associated with the membranes of cells in the hearts of Sham animals were found primarily in the cytosol fraction of MI hearts (Figure 6).

### Desmoglein-1c (DSG-1c) Is Expressed in MSCs, Enriched in MSC Exosomes, and Taken up by Ischemic Myocardium

**FIGURE 7:**
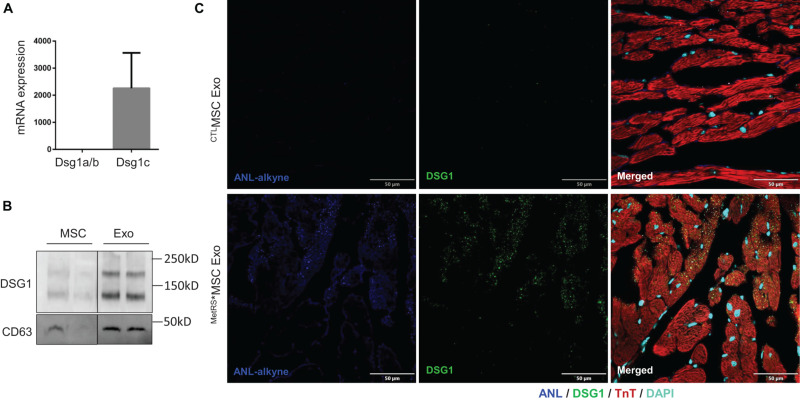
MSC exosomes contain DSG1 and deliver DSG1 to the cardiomyocytes of infarcted hearts. **(A)** mRNA levels of Dsg1 isoforms a/b and c were evaluated in MSCs via qRT-PCR and normalized to GAPDH mRNA levels. **(B)** The abundance of DSG1 protein in MSCs and MSC exosomes was evaluated via Western blot. **(C)** ^MetRS*^MSC-Exos and ^CTL^MSC-Exos were injected into the tail vein of mice 3 days after surgically induced MI. Mice were euthanized 4 h after exosome administration, and heart-tissue sections were click-stained to identify the ANL-labeled ^MetRS*^MSC exosomal proteins and immunofluorescently stained for the presence of DSG1 and troponin T (TnT) protein; nuclei were counterstained with DAPI.

The results from our click-catalyzed alkyne-agarose capture assessments indicated that desmoglein 1 (DSG-1) was more abundant in the heart than in other organs of both MI and Sham animals, and that cardiac DSG-1 levels increased in response to MI; however, to our knowledge, DSG-1 expression had not been previously reported in either MSCs or cardiomyocytes. Thus, we evaluated DSG-1 expression in MSCs via RT-PCR and in both MSCs and MSC exosomes via Western blot: our results confirmed that the DSG-1c isoform was expressed in MSCs (Figure 7A) and enriched in MSC exosomes (Figure 7B). To determine whether we could identify MSC-derived DSG-1 in the infarcted myocardium of ^MetRS*^MSC-Exo–treated mice via FUNCAT, myocardial sections were click-stained with biotin-alkyne, sequentially stained with primary biotin antibodies and fluorescent secondary antibodies, and then counterstained for the presence of DSG-1 and troponin-T (TnT). When visualized via fluorescence, a number of TnT-expressing cells were positive for both the biotin-alkyne and DSG-1 labels (Figure 7C), which confirmed that the cardiomyocytes had taken up ^MetRS*^MSC-Exo–derived DSG-1.

## Discussion

Because the benefits of stem-cell therapy for myocardial repair are primarily mediated via paracrine mechanisms, the factors produced by these cells, many of which are transported by exosomes, could serve as a potent, cell-free alternative for the treatment of patients with cardiovascular disease (Liu et al., 2018). Results from a number of preclinical studies indicate that MSCs and the exosomes produced by them are equally beneficial (Xiao et al., 2018); however, the mechanisms that direct the homing of exosomes to injured tissues, as well as the uptake and biodistribution of their cargo, remain largely uncharacterized, because techniques for distinguishing between exosomal proteins and proteins from the targeted tissues are either unavailable or inefficient. Here, we demonstrate that when MSCs are transfected with a vector coding for MetRS^∗^ and cultured in ANL-supplemented medium, the proteins produced by the transfected MSCs are selectively labeled with ANL, which can be covalently linked (via click chemistry) to alkyne-tagged reagents for subsequent visualization, identification, and isolation. Thus, when exosomes produced by the ^MetRS*^MSCs were systemically administered to mice, the protein components of their cargo could be accurately and reliably tracked, visualized, identified, and quantified in tissues from a variety of organs, including both intact and infarcted hearts. Notably, as bioorthogonal chemistry can occur in living systems without disrupting native biochemical processes (Prescher et al., 2004; Prescher and Bertozzi, 2005; Sletten and Bertozzi, 2011; Ngo et al., 2013; Han et al., 2020a), this approach can be applied to other cell populations and even extended to wholly *in vivo* investigations by generating MetRS^∗^-transgenic mice (Alvarez-Castelao et al., 2017; Han et al., 2020a).

The overall pattern of ^MetRS*^MSC-Exo protein distribution differed substantially between organs; thus, the mechanisms that govern exosome homing and the processing of exosomal proteins after uptake are likely cell- and tissue-type specific. Furthermore, our observation that a number of canonical exosome marker proteins (e.g., TSG101, Syntenin-1, ALIX, CD9, and CD81) were not present in any of the organs analyzed suggests that they are not suitable for tracking exosome uptake; whether their absence is attributable to rapid degradation in the targeted tissues or another process will be investigated in future studies. We also observed that surgically induced MI led to an increase in the abundance of exosomal proteins in cardiac tissue, which is consistent with the therapeutic mechanism of systemically delivered exosomes (Xiong et al., 2021) but has not been explicitly demonstrated previously. MI was also associated with the redistribution of numerous exosomal proteins from the membrane fraction in intact hearts to the cytosol of cells in infarcted hearts. This observation was not anticipated but likely evolves from increases in membrane permeability, because the loss of cardiomyocyte membrane integrity is a hallmark of MI, and some proposed treatment strategies target membrane dysfunction (Han et al., 2020b). Our results also indicated that the desmosome protein DSG-1c was expressed in MSCs, enriched in MSC exosomes, and taken up by cardiomyocytes in response to MI and ^MetRS*^MSC-Exo administration. DSG-1 expression has not been previously reported in cardiomyocytes; however, cardiomyocytes express DSG-2, which is critical for cardiac integrity and electric conduction. Notably, human DSG-2 mutations are associated with cardiac arrhythmia (Schinner et al., 2020), and genetic deletion of DSG-2 leads to arrhythmogenic cardiomyopathy and heart failure in mice (Pilichou et al., 2009); thus, whether the exosome-mediated transfer of DSG-1 to cardiomyocytes contributes to the benefit of MSC therapy warrants continued investigation, particularly since MSC exosomes appear to reduce the arrhythmogenicity of human cardiomyocytes (Sattayaprasert et al., 2020).

A limitation of this study is that N-terminal processing by methionyl aminopeptidases results in cleavage of the first residue during protein maturation, preventing identification of these proteins. Methionine is the first amino acid translated during protein synthesis in eukaryotes and the average methionine content of mammalian proteins is ∼2.13% (Dieterich et al., 2006). Nevertheless, N-terminal posttranslational processing and acetylation occurs in ∼30% of all mammalian proteins (Meinnel et al., 2005) and only ∼1.02% of the open reading frames of the human database (predicted to be <25 residues and incomplete sequences) do not contain a single methionine residue (Dieterich et al., 2006).

In conclusion, when exosomes were collected from MetRS^∗^MSCs that had been incubated with ANL and then systemically administered to mice, the ANL-labeled exosomal proteins could be accurately and reliably identified, isolated, and quantified from a variety of mouse organs, including both intact and infarcted hearts, via ANL-alkyne click chemistry. Thus, this bioorthogonal technique can provide crucial insights into the mechanisms that govern exosome homing and the uptake of exosomal proteins.

## Data Availability Statement

The datasets presented in this study can be found in online repositories. The name of the repository and accession numbers can be found below: European Bioinformatics Institute (EBI) PRoteomics IDEntifications Database (PRIDE) Archive, https://www.ebi.ac.uk/pride/archive/, PXD024482, PXD024483, PXD024484, and PXD024485.

## Ethics Statement

The animal study was reviewed and approved by the Institutional Animal Care and Use Committee (IACUC) of the University of Alabama at Birmingham.

## Author Contributions

EZ and GQ conceptualized the study, interpreted the data, and wrote the manuscript. EZ, YL, CH, CF, LW, WC, YD, DH, SX, YW, and JM performed the experiments and analyzed the data. BA and JM made intellectual contributions and assisted in data interpretation. All authors contributed to the article and approved the submitted version.

## Conflict of Interest

The authors declare that the research was conducted in the absence of any commercial or financial relationships that could be construed as a potential conflict of interest.
