# Supplementary material for: Visualization and Identification of Bioorthogonally Labeled Exosome Proteins Following Systemic Administration in Mice
Source: Front Cell Dev Biol. 2021 Apr 7;9:657456. doi: 10.3389/fcell.2021.657456 (PMC8058422; doi:10.3389/fcell.2021.657456)
Supplement: Supplementary file 1 [file Data_Sheet_1.PDF]

Visualization and identification of bioorthogonally-labeled exosome proteins following systemic administration in mice

Eric Zhang[1], Yanwen Liu[1], Chaoshan Han[1], Chengming Fan[1],,Lu Wang[1], Wangping Chen[1], Yipeng Du[1], Dunzheng Han[1], Baron Arnone[1], Shiyue Xu[1], Yuhua Wei[1], James Mobley[2], Gangjian Qin[1]

Supplementary Materials

Supplementary Figure S1

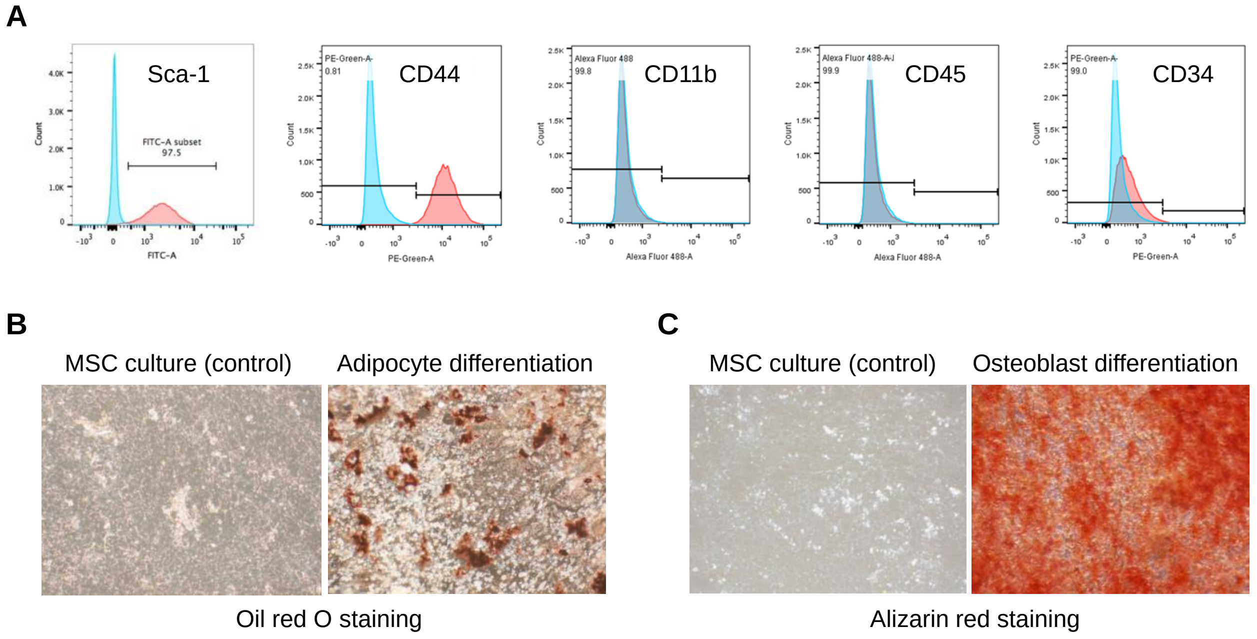

Characterization of MSCs: (**A**) FACS analysis of MSCs for cell surface markers. (**B-C**) MSCs differentiation in culture into adipocytes (**B**, *Right* panel, oil red staining, *Left* panel, control) and osteocytes (**C**, *Right* panel, alizarin red staining, *Left* panel, control). Shown are representatives of three independent experiments.

Supplementary Table S1

	Forward sequence	Reverse sequence
**For assessment of murine Dsg-1 isoform expression with qRT-PCR**		
Dsg-1c	(muDsg1abc-F) AGTGGGAGAGTTTGTAGCTA	(muDsg1c-R) CATTACATATCTGTATCTAAC
DSG-a/b	(muDsg1abc-F) AGTGGGAGAGTTTGTAGCTA	(muDsg1ab-R) ATTGTCCCCGTGCAGGTCCTC
**For generation of Lenti-CAG-MetRS^{L274G}–mCherry vector**		
5′ primer "CAG-WPRE-5"	GGCGCGCCACCGGTACGCGTGATATC AGCTGAGCTTGGACTCCTGTT	
3′ primer "CAG-LINK-3"	GATATCACGCGTACCGGTGGCGCGCC ACCGTCGACTGCAGAAT	
5′ primer "Sal1-L274G-mCherry	TGGTCGACTGGCTAGCGCCACTATGGA	
3′ primer "Age1-L274G-mCherry	TAACCGGTAACGGGCCCTCTAGACTCGAG	

Supplementary Table S2

Positive/Negative exosome marker expression	RepID (MOUSE)	Accession Number	Spectra counts
14-3-3 protein zeta/delta	1433Z	P63101	8
Alpha-enolase	ENOA	P17182	13
Annexin A2	ANXA2	P07356	37
Annexin A5	ANXA5	P48036	11
A-X actin	Actb	Q61276	24
CD81 antigen	CD81	P35762	8
CD82 antigen	CD82	P40237	4
CD9 antigen	CD9	P40240	5
Clathrin heavy chain	Q5SXR6	Q5SXR6	57
Cofilin-1	COF1	P18760	4
Elongation factor 1-alpha 1	EF1A1	P10126	12
Fructose-bisphosphate aldolase A	ALDOA	P05064	5
Heat shock cognate 71 kDa protein	HSP7C	P63017	73
L-lactate dehydrogenase B chain	LDHB	P16125	3
Lysosome-associated membrane glycoprotein 1	LAMP1	P11438	3
Programmed cell death 6-interacting protein	Alix	Q9WU78	79
Rab GDP dissociation inhibitor beta	GDIB	Q61598	2
Syntenin-1	SDCB1	O08992	28
Tetraspanin-14	TSN14	Q8QZY6	2
Tetraspanin-9	TSN9	Q8BJU2	8
Tumor susceptibility gene 101 protein	TSG101	Q61187	5
GAPDH	GAPDH	P16858	0
EEF2	EEF2	O08796	0
VCP	VCP	Q01853	0
Clathrin	Clathrin	P84091	0
Albumin	Albumin	P07724	0
